# Investigating the Relationships Between Cultural Embeddedness, Happiness, and Knowledge Management Practices in an Inter-Organizational Virtual Team

**DOI:** 10.3389/fpsyg.2020.512288

**Published:** 2021-02-12

**Authors:** Hao-Fan Chumg, Chao-Jung Huang

**Affiliations:** ^1^Department of Business Administration, National Chin-Yi University of Technology, Taichung City, Taiwan; ^2^Department of Information Management, Tamkang University, New Taipei City, Taiwan

**Keywords:** knowledge management, embeddedness theory, virtual team, happiness, *guanxi*, Confucianism

## Abstract

In light of the considerable disparity in the thinking patterns between Western and Chinese societies, this study seeks to develop a better comprehension of the influences of traditional Chinese cultural elements on knowledge management (KM) practices within contemporary Taiwanese society. To this end, the research draws on the concepts of “*guanxi*” based on the embeddedness theory concept through the lens of employees’ happiness. A qualitative case study was conducted within a virtual team (in this case, Taiwanese Farmers’ Associations (FAs)), and data were collected from both observations and 36 interviewees. Based on the resulting analysis, the findings show that individuals’ knowledge-sharing behavior results from a complex interplay of a sense of well-being, *guanxi*, and the Chinese culture rooted within such organizations. The study provides a significant insight into the cultural implications of KM strategies and offers valuable advice for knowledge managers within the context of Taiwanese society.

## Introduction

An increasing number of academics and practitioners are aware of the critical linkage between successful knowledge management (KM) practices and national cultures to maintain organizations’ sustainable development. KM is a complicated social–technological system that consists of the generation, retrieval, and dissemination of knowledge and expertise within a firm to maintain and enhance its competitiveness. Meanwhile, specific functionalities and traits of KM systems are based mainly on the beliefs inherent in the cultural backgrounds of their practitioners and designers. Prior research studies have made much of how traditional Chinese cultural factors affect individuals’ strategies to share their knowledge with others (Yan et al., 2016). However, with its fast-growing economy and the political transformations of recent years, Taiwan may be more inclined to preserve the traditional Chinese culture and Confucianism (Lin and Ho, 2009). Only a few studies have explicitly explored the impacts of Chinese culture on KM practices within Taiwanese organizations. Therefore, a fewer research in this field lacks a cultural explanation of contemporary KM practices within such Chinese organizations in Taiwan.

On the other hand, prior researchers have successfully predicted the role of employees’ happiness because of its substantial influence as an antecedent and determinant of employees’ knowledge-sharing behavior (Chumg, 2015). Nonetheless, most of the literature focusing on individuals’ happiness is heavily weighted in terms of Western countries while few studies have been conducted with regard to the Asian culture and collectivist samples. For instance, social influences may be explored through such concepts as social capital, which may be widely regarded as one of the main theories through which mechanisms affecting individuals’ sense of well-being can be studied, a sense which, in turn, influences their willingness to share knowledge (Chumg et al., 2016). However, such a theory (i.e., social capital), which is derived from a Western perspective, may be not suitable to apply to organizations within the context of Chinese society (Lisha et al., 2017). Many research studies on social relationships have only explicitly considered the context of the cultural environment where the degree to which networks are embedded is less. Recent studies have shown how *guanxi* (uniquely referring to Chinese interpersonal relationships) has a profound and all-pervasive impact in Chinese society (Zhan et al., 2018). Literarily, *guanxi* is composed of two syllables and is a Chinese phrase indicating that interpersonal relationships are a fundamental aspect of Chinese culture. The first syllable, “*guan*,” implies a door or gate and refers metaphorically to a unique circle of social networks where one within the circle is deemed to be an in-group member or vice versa (Lisha et al., 2017). The second syllable, “*xi*,” means a social network, tie, or relationship (Lisha et al., 2017). Chen et al. (2018) also show that strong *guanxi* networks positively affect the creativity of firms and this, in turn, influences entrepreneurial happiness. Hence, how to maintain, or even enhance *guanxi*, is reflected and is rooted in instrumental and affective elements in the social processes of Chinese society (Keith et al., 2016).

In order to respond to this knowledge gap, this research explores the joint effect of psychology (employees’ happiness) and sociology (*guanxi*) on KM practices in a virtual team embedded in a Chinese cultural context. It begins by exploring the relevant literature concerning the possible and complicated effects of happiness and *guanxi* on knowledge-sharing behavior in Taiwanese society. The context of the study and the sample to be investigated are then described. The research approach is outlined in detail, together with the strategies of inquiry that were chosen. The whole process is articulated in terms of the selection of the methods, the analytic tools and sampling, and the development of the instruments for the qualitative approach. Finally, the work concludes with a presentation of an overview of the research findings, together with its implications, limitations, and recommendations for future research. The research endeavors to solve the questions below:

(1) What factors influence the happiness of employees in KM practices within a virtual team embedded in Taiwanese society?
(2) How does the effect of *guanxi* on Taiwanese employees’ behaviors help to explain KM practices within a virtual team?
(3) Does the happiness of Taiwanese employees relate to the influence of *guanxi* on their knowledge-sharing behavior within a virtual team?

## Theoretical Development

### Cultural Embeddedness of *Guanxi* (Uniquely Chinese Interpersonal Networks) and Their Impact on Contemporary Taiwanese Society

Embeddedness theory, pioneered by Polanyi (1944), is an integration of economics and sociology, highlighting the importance of human economic activities which are embedded in society and institutions. Granovetter (1985) utilized such a viewpoint to study organizational theory and proposed that the trust generated by interactions among people should be regarded as a crucial decisive factor in the cost of transactions. Cultural embeddedness means the degree of people’s collective understanding of the role played in forming economic strategies and goals (Dequech, 2003). Granovetter (1992) extended and divided this concept into “structural embeddedness” and “relational embeddedness.” Relational embeddedness refers to the mutual relationships of individuals developed through the process of interactions while structural embeddedness focuses on the social characteristics of connections between people or units (Horak and Taube, 2015). In this research, embeddedness theory is employed to investigate the role of the cultural embeddedness of *guanxi* within a virtual team in Taiwanese society. The term “*guanxi*” is a specific Chinese cultural and social phenomenon that exists pervasively and exerts a strong influence in every realm of Chinese people’s lives and society; “*guanxi*” underlines the importance of trust, obligation, and reciprocity (Yen et al., 2011). Basically, the concept of *guanxi*, which was developed following Confucious’ thought (551–479 BCE), forms the modern base for Chinese cultural practices (Hwang and Staley, 2005). According to the analects of Confucius translated by Lun (1996), written by his followers who recorded his sayings and acts, he proposed three central tenets inherent in the five primary relationships of: (1) ruler and subject; (2) friend and friend; (3) husband and wife; (4) brother and brother; and (5) father and son. These tenets are loyalty, benevolence, and fulfilling responsibilities. Among the five cardinal relationships mentioned above, father and son, brother and brother, and husband and wife are involved with familial *guanxi* (Ho and Redfern, 2010). All Chinese interpersonal relationships stem from a combination, to a varying degree, of these five relationships (Hwang et al., 2009). It lies mainly in the social value of traditional Chinese collectivism, that is, someone fulfils inherent responsibilities in a previously established role to maintain harmonious interpersonal relationships (Patt-Shamir, 2006). This unique aspect of the Chinese indigenous culture is widely defined as the ubiquitous interpersonal relationships underlying high-quality social connections and the mutual exchange of favor and benefit. Hence, the vast majority of Chinese people have a strong tendency to develop, build, and maintain *guanxi* with their colleagues and view *guanxi* as being not only ingrained but also irreplaceable in Chinese society.

More simplistically, *guanxi* can be deemed to “pass the gate and get connected” (Lee and Dawes, 2005). This is mainly because, in Chinese society, lower trust and familial collectivism lead to interpersonal trust being restricted to family members; it does not extend to others. Alternatively, they tend to establish dyadic relationships with non-family members while developing social networks. Hwang (1998) further stated that, in this process of socialization, Chinese people tend to engage in activities related to others within the same *guanxi* network. Yet, a review of prior literature shows no literal translation or comprehensive understanding of the *guanxi* of the Chinese culture in Western countries (Haley et al., 2004; Lisha et al., 2017).

In Chinese society (e.g., Taiwan), there are various dimensions of *guanxi* that may have affected business performance and competitiveness (Yen et al., 2011). Recognizing the significance of *guanxi* in all aspects of Chinese society is necessary for managers of Western organizations to understand clearly how such *guanxi* is and what elements of *guanxi* exist within Chinese society. Prior literature has indicated that the *guanxi* concept has been built through four main related constructs: *ganqing*, *renqing*, *xinren*, and *mianzi* (Yen et al., 2017). Jacobs (1979) stated that *ganqing* tends to accrue following social interactions; it often occurs when people work together, cooperate, and are good to each other. *Renqing*, which is emphasized in the Confucian philosophy, reflects reciprocity. People who have good *guanxi* are especially bonded by the social obligation of reciprocity (Yen et al., 2011). Meanwhile, the progressive increase in the identities of Chinese employees positively affects the *xinren* (a Chinese word related to the level of trust) their Chinese colleagues and employees have for them (Law and Jones, 2009). Tong and Mitra (2009) indicated that *mianzi* refers to fear of losing face. These main items not only are construed as individual dimensions of *guanxi*, but also have helped in further developing a more dimensional measure of *guanxi* in this research.

### Cause–Effect Relationship Among Employees’ Happiness, *Guanxi*, and the Chinese Culture

An increasing number of academics have explored positive psychology. For example, Seligman and Csikszentmihalyi (2000) pointed out that positive psychology involves hope, satisfaction, contentment, optimism, and happiness; in short, it focuses on how to make people’s lives more meaningful and valuable. Meanwhile, the East Asian view of happiness as the realization of social harmony has a close connection with certain Asian ideas as revealed in Confucianism (Uchida et al., 2004). This could be said to correspond to the study of Buttery and Wong (1999) who stated that, in Chinese society within the doctrine of Confucius, harmony is generally thought to be an important characteristic which should be promoted and pursued.

In addition, Jacobs (1980) states that *guanxi* consists of common social identities. The concept of social identity concerns the extent to which an individual considers himself/herself as part of a social group and being a member of this group (Tajfel, 1972). The social identities of *guanxi* are close to the dimension of social integration in social well-being mentioned by Keyes (1998). The term “social integration” is an evaluation of the connection between individuals and society and groups (Keyes, 1998). In addition, integration refers to the degree to which people feel that they have something consistent with others who construct their social reality, and the degree to which they realize that they are the part of the society and community. Establishing interpersonal relationships based on trust among people can be regarded as an important element that triggers their happiness (Keyes, 1998). An additional concept prevalent in the Chinese context is *zerengan*, usually translated as a “sense of responsibility” (Davison et al., 2020). In Chinese society, family relations and the ethics can easily be copied to individuals’ workplaces (Michailova and Hutchings, 2006). Senior employees are regarded as being responsible for protecting and caring about happiness of their subordinates (Han and Altman, 2008). The benevolent behaviors shown by superiors, such as personal care for employees, forgiveness of mistakes, and comprehensive consideration of happiness of subordinates, are regarded as models of moral leadership. In turn, the subordinates who benefit from the supervisor–subordinate relationship will also be satisfied with their work (Cheung et al., 2008).

In this article, the terms “happy,” “higher positive emotion,” “happiness,” and “well-being” are all used to describe a person who feels positive emotions (as opposed to negative ones) most of time. Likewise, the research considers that Taiwanese employees’ behavioral motivations and patterns may result, at heart, from an interplay of culturally driven forces, *guanxi*, and Confucianism. Few research studies have investigated to any depth the complex interplay of employees’ happiness, *guanxi*, and Chinese cultural forces, and so this research aims to explore in depth how the abovementioned factors may contribute to individuals’ knowledge-sharing behavior through information and communication technologies (ICTs), such as video-conferencing and social networking (e.g., Facebook and Line), within a virtual team in the Taiwanese context.

### Context of the Study

The virtual team selected for this research includes the dynamic and temporary integration of Taiwanese Farmers’ Associations (FAs); these people gather together to pursue market opportunities and complete organizational tasks (Chumg, 2015). Moreover, this research defines a virtual team as a group of employees who work from disparate geographic locations and communicate with each other through various ICTs, such as email, Facebook, Line, and video conferencing services, in order to collaborate. Meanwhile, the virtual team also establishes a KM system library where members can store and retrieve their knowledge. More importantly, *guanxi* plays an important role in affecting workplace network and performance in the Taiwanese society. Hence, we propose that, in order to improve collaboration effectively, members of a virtual team build open communications and share knowledge with each other. The virtual team uses various Information Technology (IT) systems to share relevant information concerning public agricultural production and sales, as well as to disseminate knowledge about agriculture. These systems are used to look at official documents, conference-related information, training courses, project management records, the knowledge sharing of members, and related videos.

## Research Methodology

### Study Design

A qualitative approach was chosen to develop a framework based on the abovementioned research problems and the corresponding research questions. Also, given the fact that the various reasons for feeling a sense of well-being embedded in *guanxi* and for the level of knowledge-sharing behavior of employees within the intricate context of a virtual team in the Chinese society are abstruse and difficult to understand in depth, the research conducted an exploratory case study. Meanwhile, the studies involving human participants were reviewed and approved by the selected Taiwanese FAs. All participants provided their written informed consent to participate in this study.

### Participants’ Characteristics and Data Collection

Considering the importance of the selection and adoption of a research sample, this study adopts the purposive sampling method, combining intensity sampling and snowball sampling to select participants in the virtual team, which consists of the four FAs. Subsequently, in order to gather qualitative data, semi-structured, in-depth interview questions were formulated after careful discussions with two KM experts to avoid “blind spots” or preconceived opinions. In addition, Institutional Review Board (IRB) approval was obtained. Meanwhile, a pilot interview, which invited four interviewees, was conducted to evaluate the content validity and reliability of the interview questions. After that, some interview questions were rephrased or redesigned because some were not clear enough, while others were vague. Then, we confirmed the final version of the interview questions and prepared them for the chosen interviewees (see Appendix A). In addition, the workplace non-participant observation of the participants which lasted for about 4 weeks was carried out in this study because such an approach can provide an opportunity to carry out an in-depth investigation of an organization. Consequently, a total of 36 interviews were undertaken with the selected case during the period of October 2014 to June 2015, with this research being finally completed in December 2016. Most employees working within the selected virtual team had more than 20 years of working experience, and their average ages were over 46 years. Only one employee, who had been working within the virtual team for less than 10 years, was fairly new (see Table 1 for more details).

**TABLE 1:** Participants’ characteristics.

**Participants’ characteristics**	**All participants (*n* = 36)**
***Information re. the Virtual team***	
FA 1	12
FA 2	8
FA 3	6
FA 4	10
***Role***	
Vice Executive Secretary	2
Secretary	2
Manager	16
Director	9
Senior Employee	7
***Gender***	
Male	16
Female	20
***Age***	
40–45 years	6
46–50 years	10
51–55 years	10
56–60 years	8
More than 60 years	2
Mean age	51
Standard deviation	5.88
***Years of organizational working experience (years)***	
Less than 10	1
10–14	2
15–19	8
20–24	16
25–29	7
More than 30	2

*FA, Farmers’ Association.*

### Interview Procedure

All interviews were arranged and conducted in sequence. The main and the added supplementary probing questions were asked to obtain further information as required. In this regard, face-to-face, one-to-one, semi-structured interviews were regarded as being most appropriate for this research owing to the fact that the interview questions were designed to elicit each individual interviewee’s particular perspectives, attitudes, and behavior with regard to knowledge sharing in the selected virtual team; joint interviews might have risked provoking confrontation and/or conflicts of interest. In order to reduce tension, all the interviews were conducted in a natural and comfortable environment of the respondents’ choice.

### Selection and Analysis of the Qualitative Approach

This study utilized thematic analysis to analyze the qualitative data mainly because of its usefulness and flexibility in interpreting rich, detailed, and complex data (Braun and Clarke, 2006). Thematic analysis is helpful to identify, analyze, and report themes within the textual data when interpreting various research topics and then finding solutions involving problems in a real-life context. Braun and Clarke (2006) emphasized another significant advantage of thematic analysis; that is, it considers both semantic topics and potential topics. With regard to the analysis technology utilized in the qualitative method of this study, NVivo 8.0 software was selected to analyze qualitative data, because it is very beneficial to the encoding and analysis of text, sound, image, and video. Similarly, this study believed that NVivo software can systematically effectively integrate and analyze qualitative data. These are all for obtaining an overview of the flow of knowledge within the virtual team and to identify key interviewees for in-depth interviews.

### Reliability

This research improves the reliability of research methods by carefully organizing interviews, strictly coding, and constantly comparing and analyzing data. Before the interviews, the researcher actively established a good interpersonal relationship with the subjects and clarified the importance, purposes, and methods of this research in advance. Subsequently, the researcher attached importance to the perspective of intersubjectivity, openness, and flexibility to capture the diverse contextual information provided by the interviewees during the interviews.

### Validity

This study used four methods to improve the validity of its qualitative research: (1) detailed description; (2) member checking; (3) semi-structured in-depth interviews to avoid the limitations of structural interview such as lack of flexibility and difficulty in in-depth discussion of problems but also avoid the time-consuming and laborious activities of non-structural interview so as to confirm the accuracy of the qualitative results; and (4) self-reflection will address the subjectivity as a researcher associated with people and events that encounter in the field.

## Results

### Relationship Between Happiness and Knowledge-Sharing Behavior

Eight respondents shared similar views concerning helping colleagues to solve problems and seeing them continually grow; this made them feel happy and gave them a sense of well-being (see Table 2). Hence, as can be seen by the comments of the respondents, it is observable that employees’ sense of benevolence and helping colleagues to solve problems can be regarded as critical elements of well-being that make employees happier within the virtual team. Moreover, based on comments from two respondents, the findings show that employees who identify highly with the organization are likely to feel a greater sense of well-being. Six respondents showed that a greater sense of well-being, and a greater willingness to share their knowledge and help others, stemmed from them having higher levels of emotional empathy toward others. Three respondents stated that they are always enthusiastic and have a passion to share their knowledge with colleagues.

**TABLE 2:** Summary and selected quotations associated with the effects of employees’ perspectives on their happiness within the virtual team.

**Effects**	***n***	**Selected quotations**
Benevolence (*Guanxi*)	8	*The greater the sense of benevolence I have, the more happy I feel. I will feel happier and more confident when I can help my colleagues to solve problems in terms of the projects of the organisation. (Director, FA 2)*
Emotional empathy *(Guanxi)*	6	*From my perspective, happiness depends on your attitude towards others. Basically, I will not like or hate my colleagues for no reason. I always treat my colleagues kindly and see the situation from their point of view. Because of positive thinking, I feel I am a blessed person. (Manager, FA 1)*
Enjoyment of helping others	3	*I enjoy sharing my knowledge with my colleagues. When we have leisure time, such as lunch time, I always join the conversation of my colleagues to share what I know with them; I feel happy about that. (Director, FA 4)*
Stronger identification	2	*I feel very happy because this job is my ideal job, consistent with my dream, learning and family background. Moreover, the job can let me achieve my targets and the place makes me feel comfortable. (Director, FA 4)*

Eight respondents stated that achieving goals or helping colleagues to complete a project in the organization gave them a sense of benevolence and made them feel happy. Seven respondents commented on their attitude toward organizational identification in facilitating knowledge-sharing behavior. Three respondents mentioned how emotional empathy affected their knowledge behavior. For instance, one respondent stated that “… *I think I am a lucky person as compared with most people. This is mainly because sometimes I read some negative news and compare my situation with them. I think my life is better and blessed. Hence, I always like to help my colleagues in the organisation.*” Meanwhile, four respondents stated that they are always enthusiastic and have a passion to share their knowledge with colleagues.

Three respondents stated that loss of *mianzi* might have a significantly negative impact on their knowledge-sharing behavior in the virtual team. For example, one respondent said: “…*Sometimes, I will be more conservative about sharing my knowledge with other co-workers through using ICTs since I do not know whether my knowledge is necessary to them. Hence, I will not share my knowledge with my co-workers unless I think my knowledge is extremely useful to other co-workers*….” Table 3 shows the factors affecting knowledge-sharing behavior together with illustrative examples.

**TABLE 3:** Summary and selected quotations associated with factors concerning employees’ knowledge-sharing behavior within the virtual team.

**Effects**	***n***	**Selected quotations**
Benevolence *(Guanxi)*	8	*Because of my position in the organisation, I have more opportunities to meet relevant agricultural workers and to help others to solve their problems in terms of agriculture and see their continual growth. Then their gratitude makes me feel a sense of achievement and well-being. (Director, FA 4)*
Stronger identification	7	*I think we do not have difficulty in cooperating or sharing knowledge in the organisation since most colleagues have a higher level of identification with the organisation. (Manager, FA 1)*
Enjoyment of helping others	4	*I am always enthusiastic and happy to share my knowledge with my colleagues. In addition, I really hope I can help them, especially new staff. (Director, FA 2)*
Emotional empathy *(Guanxi)*	3	*I tend to share my knowledge with my subordinates. This is because I propose that I have more opportunities to obtain new knowledge and present it at conferences as compared with my subordinates, and therefore, I consider that my subordinates maybe need my knowledge. (Director, Company 4)*
*Mianzi* (Chinese culture)	3	*I think I will not actively share my knowledge with my co-workers. This is because I am not sure whether the knowledge I share is correct or not, and whether my knowledge is useful to my co-workers. Sometimes, I will also consider their feelings since I don’t know whether they need the knowledge I share or whether we have the same point of view. As a result, I prefer receiving knowledge from my co-workers rather than sharing it myself. (Manager, FA 3)*

### Relationships Among Happiness, *Guanxi*, and Knowledge-Sharing Behavior

Nine respondents expressed a strong preference for *renqing* from other colleagues within the organization (see Table 4). They considered that it is very important for them to get support from others when they needed it, and such supportive behavior on the part of their colleagues made them feel happier. *Renqing*, which is emphasized in Confucian philosophy, reflects reciprocity and is interchangeable with the Chinese word “*huibao*”. Hence, when employees have good *guanxi*, they are especially bonded by such social obligation. There were indications from 8 respondents’ comments that to have “*good ganqing*” among colleagues is a pivotal element in the sense of well-being and that this is linked to their cooperation with each other. Specifically, *ganqing* is a Chinese phrase often used to describe the quality of a relationship between two parties. The findings from the qualitative interview data showed that a total of six respondents described a significant level of *guanxi* (*Tongxiang*, *Tongxue*, *Tongshi*) among colleagues in terms of their workplace networks within the organization. Fu et al. (2006) described the term “*tong*” of “*guanxi*” as the structures of people’s personal networks in terms of common social identities, such as birthplace (*tongxiang*), educational institution (*tongxue*), and workplace (*tongshi*). These features are constructed and formed through geographic propinquity, families and organizations, and isomorphic positions in social systems. Hence, sharing social identities among colleagues seems to strengthen cooperative relationships in workplace networks, making employees happier within the organization. Two respondents highlighted the fact that all their supervisors helped and supported them whenever necessary.

**TABLE 4:** Summary and selected quotations associated with the effects of *guanxi* on employees’ happiness within the virtual team.

**Effects**	***n***	**Selected quotations**
*Renqing (Guanxi)*	9	*I keep in a good mood since our colleagues always help each other and support me when I am busy at work in the organisation. I am really thankful for their help; otherwise, I think I would be very tired without their help. (Senior Director, FA 4)*
*Ganqing* (*Guanxi*)	8	*I think most colleagues have shared memories in the organisation because most colleagues have had ‘good ganqing’ with each other for around twenty years. As we have worked hard together for a long time, we have created profound friendships with each other and deem the organisation as being an extended family. (Senior Employee, FA 2)*
*Tongxiang*, *Tongxue*, *Tongshi (Guanxi)*	6	*I have known the service department director (Respondent 17) well for 30 years since we have lived in the same area and studied in the same school from when we were very young. Hence, we are always happy to work together and share a lot of working experience within the organisation. (Secretary, FA 3)*
Supervisor–subordinate (*Guanxi*)	*2*	*My supervisor treats me as a small group member. He always helps and takes particular care of me no matter whether in public or private activities. This makes me feel respected and valued. This is mainly because we were born in the same town and studied at the same school. She introduced me to work in the organisation. (Director, FA 1)*

As shown in Table 5, four respondents stated that they might be favorably inclined to cooperate with others because of the existence of long-standing friendship and *xinren* (trust). *Xinren* can be deemed to be a significant dimension of *guanxi*, which means that there is trust, reliance, belief, and dependence existing in a relationship; yet, similar to *guanxi*, *xinren* takes time to build. Three respondents stated that having “*Ganqing shen*” among colleagues can be regarded as a driver that triggers their knowledge-sharing behavior. “*Ganqing shen*” can be translated as “*deep ganqing*,” as illustrated by Chen and Chen (2004) who explained that this involves a vast amount of emotional attachment; it has been used to explain a specific social relationship developed and built over a long period of time. Six respondents stated that their similar educational backgrounds, lifestyles, and geographic propinquity had created a compact workplace network for them within the organization. Hence, interpersonal similarity is the primary driver behind knowledge sharing within the selected virtual team.

**TABLE 5:** Summary and selected quotations associated with the effect of *guanxi* on employees’ knowledge-sharing behavior within the virtual team.

**Effects**	***n***	**Selected quotations**
*Tongxiang*, *Tongxue*, *Tongshi* (*Guanxi*)	6	…*most colleagues were born in the same geographic area so they live, study, play and work together in the organisation. Hence, I feel that most colleagues are treated as family in the organisation. (Senior Employee, FA 2)*
*Xinren* (*Guanxi*)	4	*Most of my co-workers and I have built long-term friendships so that we can mutually xinren each other as compared with new co-workers. Based on this trust-based relationship, we can always cooperate with each other to effectively enhance our work performance. (Senior Director, FA 3)*
*Ganqing* (*Guanxi*)	3	*I have known most colleagues for more or less 10 years, and cooperate with them very well since I have worked in the organisation for more than 20 years. I have ‘ganqing shen’ with them; hence, I always offer my colleagues timely help. (Senior Director, FA 4)*

### Relationships Among Happiness, Knowledge-Sharing Behavior, and Cultural Embeddedness

As shown in the data, more than half of the respondents (12) made comments about the organization as an extended family whose members are concerned for each other. As noted above, and as can be seen Table 6, it is clear that employees have a feeling of being at home and of a sense of belonging when working in the organization since colleagues are like family and their supervisor is like a parent who treats them in a very friendly way. The leaders, or heads of the virtual team, are considered to be mentors or perhaps even parent figures. Thirteen respondents shared a similar opinion and said that they had a willingness to work within the organization because the organization was relatively stable and could provide job security in Taiwan. Meanwhile, they also showed that job satisfaction and organizational commitment were highly associated with being a permanent worker. This is mainly because the job satisfaction of employees, in terms of having a stable working style and organizational commitment influences their happiness. Ten respondents revealed that relative income is an important determinant regarding the source of their happiness. Yet, three respondents highlighted the relationship between pressure of work and their happiness within the virtual team because of the higher standard of self-respect. Thus, self-respect is more strongly associated with happiness in individualistic societies than in collectivist ones. Yet, three respondents highlighted the relationship between pressure of work and their happiness within the virtual team because of the higher standard of self-respect. Another main pressure of work leading to employees’ unhappiness concerned the heads of the organization. Four respondents made specific reference to the leadership style of their bosses and their sense of well-being.

**TABLE 6:** Summary and selected quotations related to the impacts of Chinese cultural traits on employees’ happiness within the virtual team.

**Effects**	***n***	**Selected quotations**
Job stability	13	*Our organisation is very stable and almost stationary. Most employees have relatively steady, routine jobs and less pressure of work. In addition, the staff of the organisation can collect a salary 16 months per year. Hence, most employees tend to work continually in this organisation until they retire. (Senior Employee, FA 3)*
Paternalistic leadership (Chinese culture)	12	…*colleagues are like family because, in private, we can joke and we get well along with each other, like brothers and sisters. The director-general always helps and encourages us. (Senior Employee, FA 2)*
Relative income	10	*As I am a veteran, I have worked in the organisation for thirty years. All my main source of income stems from the organisation. A relatively stable income makes me feel a sense of well-being and therefore helps me raise my children. (Manager, FA 3)*
Pressure of work	3	*Sometimes I feel pressure of work and am unhappy because I need to do a lot of projects at the same time or I cannot successfully complete my job on time. (Senior Employee, FA 2)*

This is demonstrated by the response of one respondent, a manager, when explaining the relationship between his sense of responsibility and knowledge sharing. He noted: “…*As a member of the organisation, I have the zerengan to preserve, transmit and share my relevant agricultural knowledge with other staff in order to enhance the development of Taiwanese agriculture.*” One respondent who had worked in the organization for 25 years, mentioned the positive impact of job stability on knowledge-sharing behavior within the virtual team. Meanwhile, as shown by the data, one of the most significant elements of the cultural characteristics of the virtual team is that of “hierarchy,” a top-down style of structured management. Table 7 shows Chinese cultural traits and their impacts on knowledge-sharing behavior within the virtual team.

**TABLE 7:** Summary and selected quotations related to the impacts of Chinese cultural traits on knowledge-sharing behavior within the virtual team.

**Effects**	***n***	**Selected quotations**
Job stability	8	*Normally, the whole system of Taiwanese Farmers’ Associations, which is regarded as an NGO, is not like a private enterprise; we have relatively less pressure of work and don’t need to compete with other colleagues for promotion. Hence, it is not difficult for us to share our knowledge, instead of hoarding it, in such an ambiance. (Director, FA 4)*
Hierarchical organizational structure (Chinese culture)	8	*There are many so-called rules in our organisation; we all need to comply with the rules that form an invisible bureaucratic team’s culture. The organisation attaches more importance to employees who complete the mission step-by-step. (Manager, FA 3)*
*Zerengan* (Chinese culture)	6	*For me, I am in charge of two main positions. One is as a researcher and the other is as the publication division director. As the publication division director, I think I have zerengan to the relevant agricultural readers by publishing magazines which make them obtain new related agricultural knowledge. (Director, FA 4)*

### Summary

**FIGURE 1:**
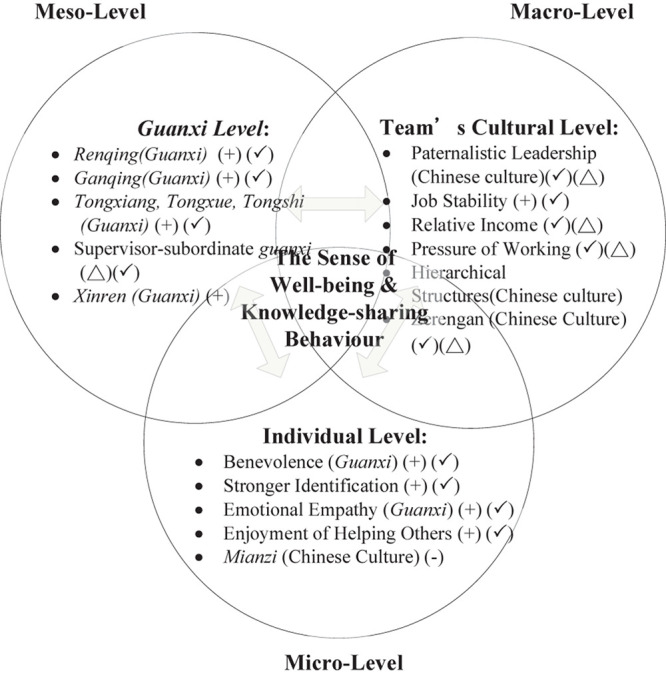
The concept of happiness and the cultural embeddedness of *guanxi* as applied in this research. (Δ) symbol means that the relationship between themes has no significant positive or negative impact on knowledge-sharing behavior; (+) symbol means that the theme (factor) positively affects knowledge-sharing behavior; (-) symbol means that the theme (factor) negatively affects knowledge-sharing behavior; (✓) symbol means that the theme (factor) affects employees’ sense of well-being.

Consequently, this study sheds light on the complex cause and effect of Taiwanese employees’ knowledge-sharing behavior through the lens of happiness and shows that it consists of three multifaceted levels: the individual level (micro level, e.g., *Mianzi*, Benevolence, Stronger identification, Enjoyment of helping others, Emotional empathy), the *guanxi* level (meso level, Chinese interpersonal relationships), and the virtual team embedded Chinese cultural level. From the perspective of the individual level, benevolence, identification, emotional empathy, and enjoyment in helping others play key roles in bridging the relationship between happiness and knowledge-sharing behavior. Moreover, from the perspective of the *guanxi* level, and this is especially so in Chinese interpersonal relationships (i.e., consisting of *renqing*, *ganqing*, *tongxiang*, *tongxue*, *tongshi*), this has a strong connection with knowledge-sharing behavior and happiness simultaneously. Finally, through the lens of the team’s culture, job stability is positively related to employees’ happiness and their knowledge-sharing behavior within the virtual team in a Chinese cultural context. Figure 1 depicts the final integrated map of the themes of the qualitative approach of the research.

## Discussion and Findings

### Key Components of the Sense of Well-Being of Chinese Employees

In order to respond to research question 1, which explored factors affecting the happiness of employees in KM practices within a virtual team embedded in Taiwanese society, thematic analysis of the qualitative approach was carried out in order to understand the determining elements of Chinese employees’ sense of well-being. The findings indicated that Confucianism, and dominant ideas in Chinese culture (e.g., Taiwan) with Confucius as their initiator, seem continually to have far-reaching influences on Taiwanese people’s thinking patterns, interpersonal behavior, occupational ethics and their sense of well-being.

At an organizational and cultural level, for most employees of the virtual team interviewed by the researcher, their jobs offered them a sense of well-being within the organization. The preferred team’s culture was a mixture of the hierarchical culture and paternalistic leadership; this involved leading by example. In the meantime, three respondents described the relationship between pressure of work and their happiness which derived from the higher standard of self-respect. This may reflect a particular Taiwanese social phenomenon, namely, multicultural characteristics, showing that individuals act or not come from self-will and independent thinking, which in turn shows that self-respect is improved to gain happiness. On the other hand, individuals may also take groups into consideration when considering the harmony of the group and the priority of group interests over individual interests, and getting along with people in harmony to obtain happiness. A total of thirteen respondents expressed strong satisfaction both with the long-term stability of their jobs and their relative income in the organization. This was because the jobs provided by the organization offered them a protected livelihood. A study conducted by De Cuyper et al. (2009) indicated that workers who were given “fixed-term or temporary contracts” reported higher levels of job insecurity than permanent workers. Moreover, most respondents considered that the paternalistic leadership caused the organization to feel like an extended family; this made them feel pleasure at work (Cheng et al., 2004). The Chinese culture emphasizes that ethical principles are the basis for leaders to influence their followers. Hence, a supervisor is expected to act as a role model for subordinates, leading by example by demonstrating their integrity and virtue (Li and Shi, 2005). Such a sense of responsibility, which is interchangeable with the Chinese expression “*zerengan*,” has been linked with assistance behavior and the tenure of a position among individuals (Davison et al., 2020). Other respondents felt appreciated and respected by certain practitioners who were helped to solve problems by themselves; this then lead them to experience the goodwill of supervisors. Through experiencing a particularly paternalistic work culture in the organization, employees felt a greater sense of well-being within it.

At an interpersonal level, employees’ who had a greater sense of *guanxi* with colleagues (*renqing*, *ganqing*, and *tongxiang*, *tongxue*, and *tongshi*) and supervisors (*supervisor–subordinate guanxi*) tended to be happier within the virtual team. *Renqing* (a Chinese phrase) is the idea of owing a favor in English (Wang, 2007). People who have good *guanxi* are especially bonded by this social obligation of reciprocity (Yen et al., 2011). Wang (2007) stated that if an exchange party receives a favor, he/she owes *renqing* to the benefactor and should be ready to make recompense in the future once circumstances permit. In order to ensure *guanxi*, a high value is placed by the Chinese on the obligation of reciprocation (Wang, 2007). There were indications from respondents’ comments that to have “*good ganqing*” among colleagues is a key element in the sense of well-being and that this is linked to their cooperation with each other. Specifically, *ganqing* is a Chinese phrase often used to describe the quality of a relationship between two parties. Jacobs (1979) stated that *ganqing* tends to accrue following social interactions; it often occurs when people work together, cooperate, and are good to each other. The findings from the qualitative interview data showed that a total of 6 respondents described a significant level of *guanxi* (*tongxiang*, *tongxue*, *tongshi*) among colleagues in terms of their workplace networks within the organization (Chen and Chen, 2004). Fu et al. (2006) and Chen and Chen (2004) described the term “*tong*” of “*guanxi*” as the structures of people’s personal networks in terms of common social identities, such as birthplace (*tongxiang*), educational institution (*tongxue*), and workplace (*tongshi*). These features are constructed and formed through geographic propinquity, families and organizations, and isomorphic positions in social systems. Hence, sharing social identities among colleagues seems to strengthen cooperative relationships in workplace networks, making employees happier within the organization. Two respondents highlighted the fact that all their supervisors helped and supported them whenever necessary. Zhang et al. (2014) claimed that employees who are engaging in supervisor–subordinate *guanxi* may feel more satisfactory and meaningful at work. This, in turn, affected their mood, especially in terms of those with whom they shared common social identities, such as birthplace (*tongxiang*), educational institution (*tongxue*), and workplace (*tongshi*). A total of nine respondents stated that they “let off some steam” by supporting their colleagues and a total of eight respondents mentioned that obtaining support from supervisors made them feel happier at work in the organization. Hence, obtaining the support and concern of colleagues and supervisors was an important spiritual pillar which encouraged respondents to continue working and which made them feel happy in the organization. Zhang et al. (2014) claimed that employees who are engaging in supervisor–subordinate *guanxi* may feel more satisfactory and meaningful at work.

In terms of the impact of positive psychology on employees’ sense of well-being, a total of six respondents highlighted the importance of positive thinking, especially in terms of having a greater sense of emotional empathy and benevolence. They considered being more empathetic made them feel a greater sense of well-being and reported feeling blessed as compared with others. This result might be said to correspond to the finding of Nonaka and Konno (1998), further indicating that feelings of greater empathy are likely to make people feel a greater sense of freedom; this leads them to share their feelings and experiences, as well as their mental model. Moreover, these findings correspond to the concepts of Chinese employees’ sense of well-being noted by Wu (1992) who asserted that an individual can obtain a Confucian-style sense of well-being through the benevolence and harmony of the group. Hence, having empathy toward others can improve employees’ happiness and interpersonal relationships. Moreover, once they have the capacity to feel compassion for others, others may feel grateful to them in response. This may help individuals who are highly empathetic to feel connected with others and experience a sense of well-being with positive results. Furthermore, stronger identification with the organization was regarded as important by a total of seven respondents; this drove them to continue to work hard. At the same time, a higher level of identification with the organization led to better performance and improved both employees’ sense of well-being and their knowledge-sharing behavior. Employees who identify highly with such aspects of the organization are more likely to create positive outcomes in terms of their work attitudes, job performance, and job satisfaction (Eatough et al., 2016).

### Chinese Employees’ Sense of Well-Being and Knowledge Sharing

To examine the roles of employees’ sense of well-being in affecting knowledge-sharing behavior, the analytic results revealed by thematic analysis showed that employees who were positive thinkers (i.e., those who found enjoyment in helping others) and had high levels of *guanxi* (derived from Confucian-style happiness such as the sense of benevolence, empathy, and identity) reported that these traits facilitated their sense of well-being and knowledge-sharing behavior. Such altruistic behavior in an individual seems likely to be positively associated with his/her sense of well-being. Hence, helping people can be regarded as a key component of on-the-job well-being for many employees. Likewise, within a stable working environment, two significantly common elements of *guanxi* and the sense of well-being (namely, *ganqing* and *tongxiang*; *tongxue* and *tongshi*) were found to be highly associated with employees’ knowledge-sharing behavior in the Taiwanese context.

### The Effect of Cultural Embeddedness of *Guanxi* on Chinese Employees’ Predisposition to Share Knowledge

For research question 2, thematic analysis was also conducted to examine if *guanxi* affects employees’ knowledge-sharing behavior. The analytic results showed that employees’ knowledge-sharing behavior, to a large extent, rests with their *guanxi* among colleagues. A total of four respondents said they would choose to share their knowledge with other colleagues, depending on whether they had taken time to build *xinren* and *ganqing* with them. This implies that *xinren* and *ganqing shen* among employees could be an important component that would give them a stronger willingness to share their knowledge in a virtual team. Previous research has shown that *xinren*, mentioned by one respondent, relates to trust (Chen and Chen, 2004). *Xinren* can be deemed to be a significant dimension of *guanxi*, which means that there is trust, reliance, belief, and dependence existing in a relationship; yet, similar to *guanxi*, *xinren* takes time to build (Yen et al., 2011). “*Ganqing shen*” can be translated as “*deep ganqing*,” as illustrated by Chen and Chen (2004) who explained that this involves a vast amount of emotional attachment; it has been used to explain a specific social relationship developed and built over a long period of time. Inkpen and Pien (2006) indicated that the existence of good friendships among partners makes them discuss and solve problems more easily.

Moreover, employees seemed to be concerned about *mianzi* within the virtual groups in a Taiwanese cultural context. Tong and Mitra (2009) indicated that *mianzi* plays an important role in Chinese society because they have a fear of losing their face. The results of this study found that, based on their upbringing in a Chinese cultural context, female employees seem to be more concerned about saving face than their male counterparts. Sometimes, therefore, they tend to hoard their knowledge instead of sharing it because of their desire to save face within a diverse working environment such as a virtual team which consists of a number of different organizations. Moreover, as noted by one respondent, having a cautious attitude, which inclined individuals not to share their knowledge, might stem from a lack of familiarity (e.g., *tongxiang*, *tongxue*, *tongshi*) with others and/or a sense that a new colleague does not “belong” to the organization. Makela et al. (2007) also indicated that interpersonal similarity is the primary driver behind knowledge sharing within the context of a multinational corporation. In other words, when a new colleague is regarded as a member of the small group, interpersonal acceptance among new and old employees may lead to fewer obstacles with regard to sharing knowledge.

### Relationships Among Employees’ Sense of Well-Being, *Guanxi*, and Their Knowledge-Sharing Behavior

For research question 3, as mentioned above, the study found that in Taiwanese society, employees’ knowledge-sharing results from the complex interplay between *guanxi* and their sense of well-being. This pervasive and fundamental element of Chinese culture, namely *guanxi* based on the Confucianism, deeply affects and informs not only employees’ psychological state but also their interpersonal behavior, which, in turn, make them feel the Chinese-style sense of well-being; this then increases their motivation to share knowledge with other colleagues. This unique feature of *guanxi* plays an important role in connecting their sense of well-being with their willingness to share knowledge with new partners within a temporary and dynamic organization in a Taiwanese context.

## Contribution to Knowledge

### Implications for Theory

From a theoretical perspective, this research concept contributes to the system of knowledge in the field of KM of virtual teams regarding knowledge-sharing behavior in the Taiwanese cultural context, in the following ways. The first theoretical contribution of the study is its exploration of the joint effect of multilevel perspectives, which was among the first. This was based on the concept of cultural embeddedness theory related to employees’ *guanxi* within the virtual team context. Many previous pieces of research have studied in detail that employees’ willingness of knowledge sharing may be affected by corporate culture development and workplace networks (e.g., Lin, 2011). However, few studies have considered what particular traits of team’s culture underlying the Taiwanese society are in need of being shaped, altered, or adjusted with an eye on the promotion of employees’ knowledge-sharing behavior or how *guanxi* influences the willingness of employees to share their knowledge; or what role employees’ sense of well-being plays under a complex virtual team context. Through this approach, it is confirmed that the factors of team’s culture (e.g., paternalistic leadership) and *guanxi* are closely intertwined and associated with employees’ happiness in a Taiwanese cultural context.

The second theoretical contribution of this study, more importantly, is profoundly exploring the importance of employee happiness in virtual groups in a Taiwanese cultural context. Walsh et al. (2018) pointed out that happier people had higher probability of offering help to their colleagues than those unhappier. Thus, the sense of well-being has the potential to play a major part in how employees respond and treat others in the workplace. This study illustrates that in Taiwanese society, employees immersed in happiness are increasingly inclined to share knowledge with their virtual team members. Many previous studies have provided clues to prove the correlation between happiness and career success (Boehm and Lyubomirsky, 2008). Existing results, however, still fail to show whether employees’ feeling of happiness is a critical motivation for their knowledge-sharing behavior, or what characteristics can bring happiness to employees in a virtual team in the context of Taiwanese culture. This study could provide a rationale for investigating happiness as a mediator in future studies about associations among workplace networks, the culture of teams, and the knowledge-sharing behavior of employees. Hence, on the basis of the current body of knowledge, the enhancement of employees’ sense of well-being is worth considering.

Third, in this study, the gap between employees’ *guanxi* within a virtual team and their behavior of sharing knowledge has been investigated in greater depth. The findings showed in Taiwanese virtual teams, employees’ positive *guanxi* tendency (e.g., benevolence, emotional empathy, *xinren*, *renqing*, *ganqing*, and supervisor–subordinate *guanxi*) could lead to a stronger willingness among virtual team members to share knowledge. In addition, the analytic results of this research revealed that a higher level of homophily among employees (e.g., *tongxiang*, *tongxue*, *tongshi*) concerning their age, social background, geographical proximity, background, and lifestyle, was regarded as the determinant that facilitated their knowledge-sharing behavior within virtual teams in the Taiwanese context. The finding of Makela et al. (2007) and Wang and Tarn (2018) demonstrated that interpersonal similarity is a key factor to improve colleagues’ willingness of knowledge sharing in multilevel groups. Therefore, employees’ higher level of homophily could be deemed as an essential element to construct a compact, friendly, co-operative and trust-based workplace network; and consequently, helps to promote knowledge sharing among employees in virtual teams in the context of Taiwanese culture.

Finally, it was found in this research that the cultural traits of virtual teams seemed to be a mixture of hierarchy-conscious and paternalistic leadership. These organizational traits might be consistent with those of traditional Chinese culture and Confucian thought. The social structure of Chinese culture is deeply dependent on the value system of Confucian (about 551–499 BC) (Tong and Mitra, 2009). They stated that Confucianism seeks to build harmony in a complex human society through creating a strongly regulated hierarchy. This may imply a need to be sensitive to hierarchy and to be able to maintain social order through micro-units, such as extended family forms within an organization. The results also show that based on Chinese culture and education, employees seem more reluctant to share knowledge with others due to their desires to save face and an innate sense of humility. The research conducted by Tong and Mitra (2009) explained the reluctance of Chinese citizens to share knowledge that they have grown up under the influence of traditional Chinese values, such as modesty, competitiveness, and fear of losing *manzi*. To continually maintain the competitive advantage of traditional industries in the era of the knowledge economy, organizations still require employees, not only to develop new technical knowledge but also to apply and share their new knowledge in an appropriate way. In the results of this study, we found that knowledge sharing stems from the top of the virtual team hierarchy. Hence, to cultivate the intrapreneurial spirit in employees at all levels of a virtual team, it seems to be a good idea for them to share their knowledge with other employees (Camelo-Ordaz et al., 2011; Chan et al., 2017; Alessio et al., 2019). Therefore, the existing relevant research provides researchers and practitioners with a clear understanding of the current situation of knowledge-sharing behavior of employees in virtual teams under Chinese (e.g., Taiwanese) cultural background, and to get a more detailed understanding of the implications of Taiwanese cultural values and its impact on KM practice.

### Implications for Practice

In last few years, Western companies have begun to pay more attention to understanding Taiwanese thinking patterns, their behavioral characteristics and businesses in order to understand the discrepancy between Taiwaneses and Westerners so to bridge the gap between Taiwanese thinking and global knowledge. The findings may have crucial managerial implications for the top Western and Eastern managers who are keen to enhance employees’ performance behavior concerning knowledge sharing in such organizations in a Taiwanese context in order to improve their competitiveness, and ultimately achieve more sustainable operations. First, at an organizational level, this study showed that, compared with Western organizations which focused on task-oriented management procedures, Taiwanese organizations play an important role in managing employees’ indirect relationships and emotional concerns. Furthermore, the paternalistic and “leading by example” style of leadership of the Chinese culture deeply affects employees’ happiness which then improves their intention to share knowledge through ICTs. Moreover, a strong feeling of the team’s stability provides them with continuous work; such characteristics of team’s culture make employees feel good. Therefore, managers of virtual teams should pay the same attention to providing an appropriate team’s culture (e.g., paternalistic culture), which can enhance employees’ sense of belonging, as well as pay relatively higher wages according to individual performance, so as to improve their happiness in virtual teams.

Second, in terms of Taiwanese social networks, the complex concept of *guanxi* has been carefully examined, and the results of qualitative interviews further consolidate this concept. The results of qualitative interview showed that the existence of friendship, involving long-term established interpersonal *xinren*, social support from co-workers and superiors, and common goals based on social similarity (e.g., *tongxiang*, *tongxue*, *tongshi*), may be an essential part to make employees feel happier and consequently enhance their willingness of knowledge sharing under the background of selected virtual teams. These aspects constitute the premise of employee happiness, which is consistent with the characteristics of employee workplace network. However, in light of the fact that the virtual team is composed of various organizations whose employees seem to be asked for building cooperative relationships in the short term, virtual teams’ managers must pay more attention to the social background of groups of members in order for them to make warm and meaningful connections with other employees as quickly as possible.

Third, the results showed that individuals with having the elements of happiness [e.g., being glad to help others, the feeling of empathy (*guanxi*), positive relationships with others, identification with the virtual team, a sense of benevolence (*guanxi*), and involvement in the emotional atmosphere of a paternalistic team’s culture] were more willing to share knowledge than their less happy peers. Yet, the study must recognize that employees’ happiness is not the only element that makes them to share knowledge with other members. Similarly, negative feeling or less happiness should not be ignored, because each emotion has its own purpose and influence. Our findings showed that happiness played a critical role in influencing the willingness of employees’ knowledge sharing in chosen virtual teams. Therefore, managers can arrange appropriate agricultural, marketing, and operation management training courses to enhance employees’ identification. They could also offer year-end benefits, such as a festival bonus; encourage employees to engage in cooperative tasks or personal social activities; improve employees’ satisfaction with their working environment; and safeguard employees’ welfare to enhance their knowledge-sharing behavior. These initiatives could ultimately increase the virtual team’s effectiveness and might be useful regarding their contribution to industrial organizational psychology.

## Limitations

The results and findings of the qualitative approach of this research offer a detailed and meaningful insight into the interconnectedness of each main construct. The relationships concerning why employees had or did not have a willingness to share their knowledge within the virtual teams in a Taiwanese cultural context were more complex than had been expected. The research sought to improve and deepen the understanding of the essential effects of employees’ happiness and *guanxi* on their knowledge-sharing behavior within the more complex context of the virtual team using the rich and meaningful descriptions of the experiences of respondents; this would have been impossible with any other research design. Even though this exploratory case study is not particularly generalizable, as noted in the initial development of the theory, it could offer a basis for comparison when studying other cases; it could also provide evidence that could be utilized by others when establishing a virtual team. A future study could benefit from incorporating a mixed-method design since a quantitative aspect to the study could further help in analyzing and interpreting the results. Consequently, it would be interesting for both Western and Eastern managers, as well as researchers, to study the long-term impact of the integration of a virtual team on Taiwanese society which is now part of this new operational mechanism.

## Data Availability Statement

The datasets generated for this study are available on request to the corresponding author.

## Ethics Statement

The studies involving human participants were reviewed and approved by the selected Taiwanese Farmers’ Associations (FAs). All patients/participants provided their written informed consent to participate in this study.

## Author Contributions

Both authors listed have made a substantial, direct and intellectual contribution to the work, and approved it for publication.

## Conflict of Interest

The authors declare that the research was conducted in the absence of any commercial or financial relationships that could be construed as a potential conflict of interest.

## Appendix A

(1) An Individual’s Attitude Toward Knowledge Sharing

1. How would you describe knowledge-sharing behavior among employees in your virtual team?
2. In what circumstances are you most likely to share knowledge in your virtual team?
3. Can you describe what kind of knowledge you share in your virtual team? How and why might you share your knowledge?
4. What are your thoughts about using Information and Communication Technology to share knowledge?

(2) Your Workplace Networks

1. How would you describe interpersonal relationships in your virtual team?
2. How would you describe your own behavior in terms of trust, collaboration, and supporting others at work?
3. How does trusting others, having shared goals, and collaborating at work influence your own performance? What benefits have you observed for yourself and for others in such practices?

(3) Your Sense of Well-Being

1. How far do you feel a sense of well-being when you are working in your virtual team? Why do you think this is?
2. In what ways do you think your colleagues and the culture of your virtual team affect your own sense of well-being? (Increased)
3. How important do you think the sense of well-being is in your organisation?
4. How does your sense of well-being influence your working performance in your virtual team? Can you describe any specific instances?

(4) Team Culture

1. Please could you describe your virtual team’s culture?
2. Does your virtual team’s culture impact on your knowledge sharing? Please explain how and why you think this is.
3. How does your virtual team encourage you to share knowledge?
